# RMTF-Net: Residual Mix Transformer Fusion Net for 2D Brain Tumor Segmentation

**DOI:** 10.3390/brainsci12091145

**Published:** 2022-08-27

**Authors:** Di Gai, Jiqian Zhang, Yusong Xiao, Weidong Min, Yunfei Zhong, Yuling Zhong

**Affiliations:** 1 School of Software, Nanchang University, Nanchang 330047, China; 2 Institute of Metaverse, Nanchang University, Nanchang 330031, China; 3 Jiangxi Key Laboratory of Smart City, Nanchang 330031, China; 4 School of Mathematics and Computer Science, Nanchang University, Nanchang 330031, China

**Keywords:** brain tumor segmentation, mix transformer, convolutional neural network, overlapping patch embedding mechanism

## Abstract

Due to the complexity of medical imaging techniques and the high heterogeneity of glioma surfaces, image segmentation of human gliomas is one of the most challenging tasks in medical image analysis. Current methods based on convolutional neural networks concentrate on feature extraction while ignoring the correlation between local and global. In this paper, we propose a residual mix transformer fusion net, namely RMTF-Net, for brain tumor segmentation. In the feature encoder, a residual mix transformer encoder including a mix transformer and a residual convolutional neural network (RCNN) is proposed. The mix transformer gives an overlapping patch embedding mechanism to cope with the loss of patch boundary information. Moreover, a parallel fusion strategy based on RCNN is utilized to obtain local–global balanced information. In the feature decoder, a global feature integration (GFI) module is applied, which can enrich the context with the global attention feature. Extensive experiments on brain tumor segmentation from LGG, BraTS2019 and BraTS2020 demonstrated that our proposed RMTF-Net is superior to existing state-of-art methods in subjective visual performance and objective evaluation.

## 1. Introduction

In recent years, the incidence of brain tumors has been increasing, and the higher mortality rate greatly threatens human health and safety. Along with the development of radiological imaging technology, the preoperative diagnostic evaluation of such diseases is playing a significant role in the clinical process. Nevertheless, manual labeling of brain tumor lesions by physicians alone is time-consuming and requires a high level of diagnostic experience, and the accuracy of labeling tumor lesions still needs to be considered. In contrast, the utilization of computer-aided medical technology for tumor diagnosis is not only convenient and fast, but also less dependent on cumulative experience, which has broad development prospects and practical significance [1,2,3,4].

MRI is a commonly performed technique in the field of radiological imaging. Because of its advantages of no damage, no ionizing radiation, and high contrast in soft tissue imaging, it has become the imaging method of choice for diagnosing and treating brain tumors. There are four standard parametric modalities frequently adopted in MRI for glioma diagnosis, including T1-weighted MRI, T2-weighted MRI, gadolinium contrast-enhanced T1-weighted MRI (T1c), and fluid-attenuated inversion recovery (Flair) [5]. Since the morphological response of different tumor tissues in MRI is contrasting, the corresponding internal anatomical tissues are imaged differently in disparate parameter modalities. In general, MRI images in the T1 modality provide the best resolution to describe the anatomical structures to distinguish healthy tissues, while MRI images in the T2 modality can depict the cystic areas that produce high brightness signals. Obviously, MRI is a multifaceted imaging technique that can display the anatomy of brain tissue, the spatial location of lesions, and their interrelationships. It can provide various information for lesion analysis and diagnosis by selecting the appropriate parameters according to clinical needs [6].

Recently, a large amount of research has been devoted to medical image segmentation. From this point of view, deep-learning techniques have caught up with machine-learning techniques in the field of medical image segmentation. Some classical convolutional neural networks (CNNs), such as VGG [7], Resnet [8], and DenseUnet [9], have successfully performed in a variety of computer vision tasks and continue to exhibit breakthroughs in performance. The rapid advancement of CNNs has allowed for the development of a large number of downstream tasks in computer vision to be fully developed [10,11,12]. Medical image segmentation has developed at high speed after the application of a fully convolutional network (FCN) [13] and U-shaped network structure (Unet) [14]. The proposal of excellent network structures such as V-net and DenseUnet has made deep convolutional networks move from theory to practice. Researchers have since focused on adding a self-attentive mechanism and gate-keying mechanism to tackle the issue of severe spatial information loss in a U-shaped network structure [15]. Unfortunately, the problem of boundary information loss due to a large number of convolutional and pooling operations in U-shaped networks is frequently neglected in the research process, which is a non-negligible problem for tumor segmentation.

Inspired by the significant success of transformers in natural language processing [16,17], a large number of researchers have endeavored to transfer these transformers into computer vision. Concretely, Vit [18] first introduced a transformer to computer vision tasks, outperforming the segmentation performance of CNNs dramatically, but its large parameters allow it to be adapted only to 2D segmentation tasks. Later, Le-Vit [19], the Swin transformer [20], and other network structures were updated on Vit, trying to reduce the number of model parameters to allow the transformer to be used for 3D segmentation tasks. However, the extensive use of transformers in this network has led to the problem of severe interference of semantic information by background information. Recently, researchers have presented the pyramid vision transformer (PVT) [21] and Segformer [22], which fused pyramid structure with transformer for extracting multi-scale features, allowing a brand-new level of segmentation performance of medical images [23]. Although this idea of fusing multiple modules is novel, the local and global information of segmented images is imbalanced.

In this paper, we propose an image segmentation method called RMTF-Net, which uses an encoder–decoder structure. RMTF-Net contains a residual MiT encoder that combines a mix transformer (MiT) and residual convolutional neural network (RCNN) structures to obtain feature information with a balance of local and global features. Due to the MiT applied in the residual MiT encoder, the encoder can work with the global attention feature during encoding. Simultaneously, the overlapped patch embedding of the MiT well protects the edge information of the patches. In the feature decoder of the RMTF-Net, we designed a GFI (global feature integration) module to re-fuse the feature information extracted from the encoder. The experimental results demonstrate that the GFI module is able to enrich the contextual information using the global attention mechanism. Finally, the feature mapping is accordingly attached to the decoder of the same size via a jump connection. The main contributions of this work are as follows:

1. We propose a novel end-to-end framework to segment brain tumors, namely RMFT-net.
2. We design a mix transformer module to reduce the interference of irrelevant background information on the segmentation results.
3. We devise a global feature integration module to enrich the context and incorporate global attention features.
4. The proposed model achieves excellent segmentation results on LGG, BraTS2019, and BraTS2020 datasets.

## 2. Related Work

### 2.1. Brain Tumor Segmentation-Based Medical Image Segmentation Method

The brain tumor is one of the deadliest brain diseases. The study of brain tumor segmentation is significant for the early diagnosis of brain tumors, which substantially improves the probability of patient recovery. Over the years, many researchers have proposed many effective segmentation algorithms based on deep-learning algorithms or machine-learning algorithms to solve this problem. In the machine-learning stage, various clustering algorithms, such as the K-neighborhood algorithm [24], K-means algorithm [25], the Perceptron algorithm [26], and so on, are based on one principle expressing similarity within a class and exclusion between classes. These algorithms can achieve certain classification effects, but they are far from the standard required for semantic segmentation. In the deep-learning stage, Long et al. proposed the FCN network [13], which can achieve end-to-end network training and accept images of arbitrary size as input. FCN became the cornerstone of deep learning to solve the segmentation problem. Since then, numerous studies have been conducted to improve FCNs from different perspectives, specifically enhancing contextual links [27,28,29], adding boundary information [30,31,32,33], etc. These approaches were proposed to boost the performance of brain tumor segmentation. Nevertheless, they made the resulting framework more complex and significantly more time-consuming for experimentation. Further methods have since demonstrated the effectiveness of the U-shaped network structure.

### 2.2. U-Shaped Network Structure-Based Medical Image Segmentation Method

Along with the proposal of a fully convolutional network FCN, Roneberg et al. [14] designed the U-shaped network (U-Net) framework for medical image segmentation. It had made significant breakthroughs in cell segmentation and various organ segmentation, and thus has been widely used in a variety of tasks. Researchers have proposed many variants based on it. Specifically, Fausto et al. [34] advanced the V-net network, which introduced an advanced objective function that can handle the imbalance between the number of foreground and background voxels. Li et al. [9] applied the DenseUnet network based on Unet, which can jointly optimize intra-slice representation and inter-slice features by a hybrid feature fusion (HFF) layer and made some progress in the segmentation task. Ozan Oktay et al. [35] proposed Attention-Unet, which developed a gating mechanism to implicitly suppress irrelevant regions. In recent years, various ideas have been proposed to address the problem of continuous pooling and convolution operations in the Unet structure leading to the loss of some spatial information. For example, the CEnet discussed by Gu et al. [36] can capture abundant high-level information and preserve spatial information. Based on such inspiration, some interdisciplinary disciplines of image segmentation have also invested much research in this direction [37,38,39]. Along with the widespread use of U-shaped networks, the problem of boundary information loss due to extensive convolution and pooling operations has become increasingly serious.

### 2.3. Transformers-Based 3D Medical Image Segmentation Method

Transformers were originally proposed by Parmer et al. [16,40] for machine translation and have subsequently been developed. Their immense success in NLP has been a major source of inspiration for researchers in computer vision. The proposal of ViT [18] has made substantial development in image classification tasks by directly applying transformers with global self-attentiveness to the input image. Compared with the traditional CNN, pre-training on large datasets is a major drawback of ViT, which leads to a magnitude increase in experimental time consumption. Therefore, in subsequent studies, researchers have continued to refine ViT and have repeatedly proposed network structures, such as DeiT [41], Swin [20], and Le-ViT [19]. Some of these studies attempted to apply the transformer structure for medical image segmentation. For example, the TransUnet network constructed by Chen et al. [42] addressed the problem that Unet exhibits limitations in explicitly modeling long-term dependencies. The PVT devised by Wang et al. [21] reduced the memory cost and employed a gradually shrinking pyramid structure to decrease the computational effort for large feature images. Traditional networks have difficulty in balancing local and global information, which leads to a large amount of information not being used rationally.

## 3. Methodology

**Figure 1:**
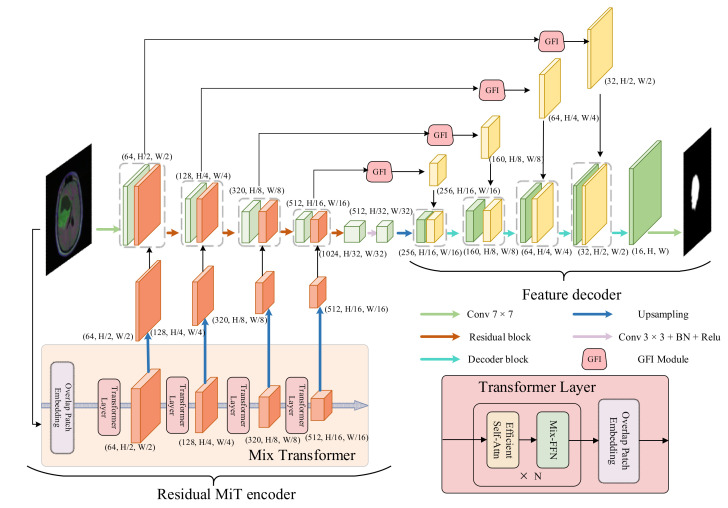
Overview of the RMTF-Net.

In this section, we introduce our proposed model in detail. The overview of our proposed model (RMTF-Net) is shown in Figure 1. The RMTF-Net contains a residual MiT encoder and a feature decoder. Concretely, the residual MiT encoder is recommended in Section 3.1, the feature decoder is presented in Section 3.2, and the hybrid loss is introduced in Section 3.3.

### 3.1. Residual MiT Encoder

The residual MiT encoder incorporates a mix transformer (MiT) and RCNN. The MiT utilizes efficient self-attention to extract multi-scale global attention features. These features provide both high-resolution coarse features and low-resolution fine-grained features. The RCNN encodes features from local to global while gradually extending the receptive field [43]. We further design an advanced parallel fusion strategy to integrate the multi-level feature maps with the same resolution extracted from both encoders.

In the encoding process, the MiT initially employs the patches divided by the original image to get four multi-scale feature maps with 1/4,1/8,1/16,1/32 resolution of the input image. After doubling the resolution of the feature maps by up-sampling, we feed these up-sampled feature maps hierarchically into the RCNN. At the same time, the RCNN splices the feature map output from the previous block with the feature map from the MiT at each residual block, where both feature maps have the same resolution. The spliced feature map is then applied as input to the next residual block. This allows the encoder to enjoy the benefits of both encoders and obtain global–local balanced features. Furthermore, the inclusion of global attention provided by MiT reduces the interference of irrelevant background information with semantic information during encoding.

#### 3.1.1. Mix Transformer

The mix transformer is composed of a single overlapped patch embedding module and four transformer layers. Each transformer layer also contains an overlapped patch embedding module. There are two other advanced modules in the transformer layer: efficient self-attention and Mix-FFN. 

During the process, the MiT generates multi-level and multi-scale features from the original image. Given an image H×W×3, MiT performs patch embedding to obtain four pyramidal feature maps. The *i*-th feature map *M_i_* with a resolution of $\frac{H}{2^{i+1}}\times \frac{W}{2^{i+1}}\times C$, where i∈{1,2,3,4} and *C_i+1_* larger than *C_i_*.

**Figure 2:**
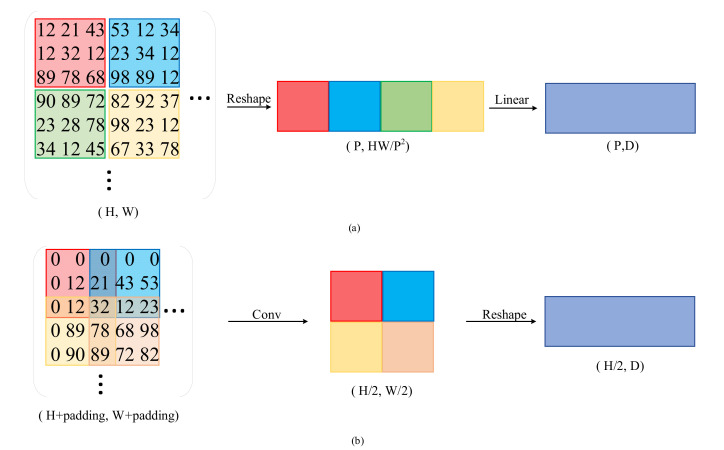
Difference between non-overlapped and overlapped patch embedding; (**a**) diagram of the non-overlapped embedding; (**b**) diagram of the overlapped embedding.

Overlapped Patch Embedding: Unlike the non-overlapped patch embedding used by ViT [18] to preserve the local continuity around patches, we propose a patch embedding strategy. The difference between them is shown in Figure 2. The overlapped patch embedding gradually shrinks the hierarchical features to obtain pyramid feature maps by merging the overlapped patches. It defines *K*, *S*, and *P*, which are similar to the parameters of convolution, where *K* is the patch size, *S* is the stride between two patches, and *P* is the padding size.

Efficient self-attention: MiT uses a sequence reduction process to decrease the complexity of the self-attention mechanism. In the traditional multi-head self-attention mechanism [16], the estimated self-attention is as follows:

(1) $$\mathrm{Attention}(Q,K,V)=\mathrm{Softmax}\left(\frac{QK^{T}}{\sqrt{d_{head}}}\right)V$$

where each of the head’s *Q*, *K*, and *V* have the same dimensions *N × C* with *N = H × W*. MiT utilizes the sequence reduction process introduced in [21] to reduce the sequence of length *N*. This process applies a reducing ratio *R* to shorten the length of the sequence as follows:

(2) $$\begin{array}{l} \hat{X}=\mathrm{Reshape}\left(\frac{N}{R},C\cdot R\right)(X)\\ X=\mathrm{Linear}(C\cdot R,C)(\hat{X}) \end{array}$$

where the X is the sequence that needs to be shortened, refers to reshaping X into a sequence of size $\frac{N}{R}\times C\cdot R$, and $\mathrm{Linear}(C\cdot R,C)(\hat{X})$ means to a linear layer with C⋅R as input dimension and *C* as output dimension acting on the output $\hat{X}$ of the reshape operation. As the length of the sequence decreases from *N* to $\frac{N}{R}$, the complexity of the self-attention mechanism is $O\left(\frac{N^{2}}{R}\right)$. The *R* from stage-1 to stage-4 is (64,16,4,1).

Mix-FFN: The fixed resolution of position encoding leads to the performance degradation of semantic segmentation tasks. To solve the mentioned problem and consider the effect of zero padding on the leak location information [44], it applies a Conv in the feed-forward network (FFN), which is named Mix-FFN, to offer positional information to Transformers. The Mix-FFN can be phrased as follows:

(3) $$x_{\mathrm{out}\;}=\mathrm{MLP}\left(\mathrm{GELU}\left({\mathrm{Conv}}_{3\times 3}\left(\mathrm{MLP}\left(x_{in}\right)\right)\right)\right)+x_{\mathrm{in}\;}$$

where $x_{\mathrm{in}\;}$ is the output of the self-attention mechanism, MLP(·) refers to the multilayer Perceptron procedure, ${\mathrm{Conv}}_{3\times 3}\left(\cdot \right)$ refers to a convolution with a kernel of size 3×3, and the GELU(·) refers to the GELU function.

#### 3.1.2. Parallel Fusion Strategy

We argue that serially fusing the Transformers and CNNs result in a loss of accuracy on account of the resolution of the feature map extracted by CNNs being too small for transformers to extract global attention features. Therefore, we introduce a parallel fusion strategy to merge the transformers and CNNs.

Initially, we double the resolution of the feature maps obtained by MiT, then before each residual block of the RCNN, we concat the feature map to be input into with the feature map output from the MiT at the same resolution. This move will introduce global attentional features into the network while supplementing the global features of RCNN. Moreover, the RCNN encoder employs the residual block to hierarchically encode the feature from local to global. The inclusion of residual connections allows the network to deal with gradient disappearance and gradient descent during training, which can accelerate network convergence. 

### 3.2. Feature Decoder

The feature decoder concludes the decoding process from high-level features to segmentation masks. It contains both GFI and decoder blocks. Similar to U-shaped networks [14], we concat the feature map output by the decoder block with the same resolution feature map output by the GFI module. This can ensure the correctness of the recovered image by avoiding the loss of some fine details via simple up-sampling during the restoration process.

**Figure 3:**
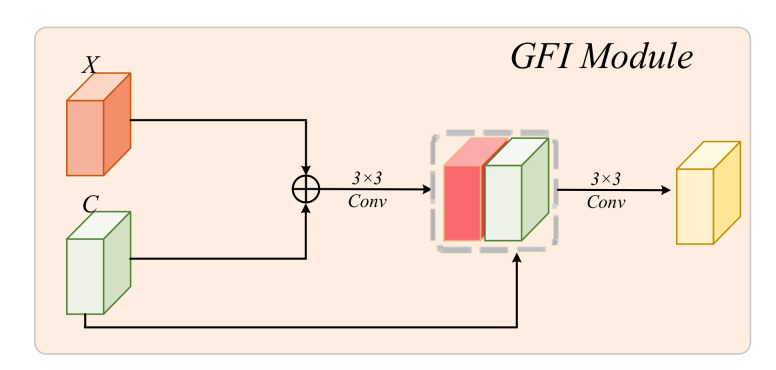
Global feature integration module.

Global Feature Integration Module: To effectively counteract feature loss during decoding of the network, we propose a global feature integration (GFI) module to enrich the context. In addition, it has the ability to balance the semantic and detailed information representations of the skip connection feature map. The architecture of the GFI module is shown in Figure 3, and it can be explained by the following equation:

(4) $$\mathrm{GFI}\left(X,C\right){=\mathrm{Conv}}_{3\times 3}([C{;\mathrm{Conv}}_{3\times 3}(X+C)])$$

where the ${\mathrm{Conv}}_{3\times 3}\left(\cdot \right)$ refers to a convolution with a kernel of 3×3, and [⋅;⋅;…] indicates the concat operation. To balance the feature redundancy between the two feature maps, the output channel of ${\mathrm{Conv}}_{3\times 3}\left(\cdot \right)$ is the same as the channel of *X*. This block can highlight the similarities between the feature maps obtained by MiT and RCNN via addition and further extract the fused features between the global attention features obtained by MiT and the local features obtained by RCNN. It can also add fused global–local features to the skip connection to enrich the contextual features available to the decoder.

Decoder block: Each decoder block includes an up-sampling procedure and two convolution blocks consisting of a 3 × 3 convolution, a BN layer, and a RELU function, which can be expressed in the following formula:

(5) $$X_{\mathrm{out}}=\mathrm{RELU}\left(\mathrm{BN}\left({\mathrm{Conv}}_{3\times 3}\left(\mathrm{RELU}\left(\mathrm{BN}\left({\mathrm{Conv}}_{3\times 3}\left({\mathrm{Upsampling}(X}_{\mathrm{in}})\right)\right)\right)\right)\right)\right)$$

where $X_{\mathrm{in}}$ and $X_{\mathrm{out}}$ refer to the input and output feature maps of each decoder block, Upsampling(·) refers to the up-sampling operation with a scale factor of 2, ${\mathrm{Conv}}_{3\times 3}\left(\cdot \right)$ refers to a convolution with a kernel size of 3, BN(·) denotes the batch-norm procedure, and RELU(·) refers to the RELU function.

After the upward feature reduction of the four decoder blocks, we use convolution with a kernel size of 7 to obtain the segmentation mask.

### 3.3. Hybrid Loss

To promote the network in a balanced way, we design a hybrid loss function consisting of Dice loss, binary cross entropy loss and SSIM loss [45]. We set the Dice loss to be $L_{1}$, the binary cross entropy loss to be $L_{2}$ and the SSIM loss to be $L_{3}$. The hybrid loss function *L* can be expressed as:

(6) $$L=\alpha L_{1}+\beta L_{2}+\gamma L_{3}$$

#### 3.3.1. Dice Loss

The Dice loss function is regularly exploited in image segmentation to measure the similarity of two images, which is given by:

(7) $$L_{1}=\frac{2\sum_{i}t_{i}e_{i}}{\sum_{i}t_{i}+\sum_{i}e_{i}}$$

where *i* denotes a pixel of two input images, *t_i_* expresses whether the current pixel point is that semantic pixel in the ground truth, and *e_i_* indicates whether the current pixel point is classified as a semantic pixel in the predicted image. Dice loss has a satisfactory response to image similarity in terms of region and has brilliant performance in scenarios with a severe imbalance between positive and negative samples, so we choose it as the main loss function of our hybrid loss.

#### 3.3.2. Binary Cross Entropy Loss

The binary cross-entropy loss function is a common loss function for binary classification problems. It is a convex optimization function that facilitates the use of gradient descent to find the optimal value, while being able to evaluate the subtle differences between the two images. Mathematically, the specific formula is as follows:

(8) $$L_{2}=\frac{-\left(\sum_{i}\left(t_{i}\times \log\left(e_{i}\right)+\left(1-t_{i}\right)\times \log\left(1-e_{i}\right)\right)\right)}{N}$$

where *N* refers to the total number of pixel points, and *i* and *t_i_* are the same as in Dice loss.

#### 3.3.3. SSIM Loss

SSIM loss, or structural similarity loss, measures the similarity between two images by brightness, contrast, and structure. The SSIM loss allows the model to acquire higher-quality images. The expression of SSIM is calculated as follows:

(9) $$\mathrm{SSIM}\left(I_{1},I_{2}|\omega \right)=\frac{\left(2{\bar{\omega }}_{1}{\bar{\omega }}_{2}+C_{1}\right)+\left(2\sigma_{\omega_{1}\omega_{2}}+C_{2}\right)}{\left({\bar{\omega }}_{1}^{2}+{\bar{\omega }}_{2}^{2}+C_{1}\right)\left(\sigma_{\omega_{1}}^{2}+\sigma_{\omega_{2}}^{2}+C_{2}\right)}$$

where $\omega_{1}$ and $\omega_{2}$ are the patch images of $I_{1}$ and $I_{2}$, respectively, ${\bar{\omega }}_{1}$ and ${\bar{\omega }}_{2}$ are the average of the pixel values of the images $\omega_{1}$ and $\omega_{2}$, respectively, $\sigma_{\omega_{1}\omega_{2}}$ is the covariance of images $\omega_{1}$ and $\omega_{2}$, and $\sigma_{\omega_{1}}$ and $\sigma_{\omega_{2}}$ are the variances of $\omega_{1}$ and $\omega_{2}$, respectively. The larger the SSIM value of two images, the greater the structural similarity of the two images. SSIM is used as a loss function, we take:

(10) $$L_{3}=1-\mathrm{SSIM}\left(I_{s},I_{g}\right)$$

where $\mathrm{SSIM}\left(I_{s},I_{g}\right)$ refers to the average of the SSIM of all windows of images *I_s_* and *I_g_*.

## 4. Experiment

### 4.1. Dataset

The experiments are based on three brain tumor segmentation datasets, including LGG, BraTS2019, and BraTS2020.

The LGG dataset is mentioned in [46,47], which contains brain MR image slices from 110 low-grade glioma (LGG) patients. The MR images of the LGG were sourced from The Cancer Imaging Archive and The Cancer Genome Atlas. After processing the dataset, we obtained a total of 1311 images for the experiment, and randomly selected 1049 of them for training and 262 for testing.

The BraTS2019 and BraTS2020 are both datasets provided by the BraTS challenge, which asked participants to evaluate a method for semantic segmentation of brain tumors by using a 3D MRI dataset with Ground Truth. Specifically, the BraTS2019 dataset contains 3D MR images of 335 patients with brain tumors. After slicing the 3D MR images and their corresponding labels, we filtered out 10,047 pairs of these images and randomly selected 8038 for training and 2091 for testing. The number of 3D MR images with brain tumors in dataset BraTS2020 was more than in the dataset BraTS2019, which included 369 cases. After slicing and filtering the 3D images of the BraTS2020 dataset, we obtained 10,945 slices with labels. Then, we stochastically chose 8756 of them for training and 2189 for testing.

### 4.2. Implementation Details

We implemented the RMTF-Net based on Pytorch. During the training process, we used the Adam optimizer with a learning rate of 0.0001 and weight decay of 0.00001 to gradually optimize parameters. For the experiment, we resized all of the 2D images in the dataset to a uniform size of 256×256, and we applied an NVIDIA TITAN GPU to accelerate our experiments. The batch size parameter for each experiment was 18. For each experiment on the LGG dataset, we iterated 100 times; for the BraTS2019 and BraTS2020 datasets, we iterated 30 times.

### 4.3. Evaluation Metrics

In order to evaluate the performance of the model comprehensively and precisely, five evaluation metrics were chosen to measure the results in various aspects, including the Dice coefficient (Dice), intersection over union (IoU), weighted F-measure (wFm), enhanced-alignment metric (Em), and structure-based metric (Sm).

These evaluation metrics reflect the strengths and weaknesses of different aspects of the model. The Dice and IoU metrics were used to evaluate the similarity between the pixel points of two image collections. The wFm metric was applied by alternately calculating the accuracy, and it extended the four basic quantities Tp, Tn, Fp, and Fn to real values. It assigns different weights to the errors generated at different locations according to the neighbor information, and thus highlights the target part of the evaluation by weighting. The Em metric can reflect both the image-level statistical information and the local pixel-matching information between two image collections. The Sm metric is a type of reconciliation index, which can simultaneously be oriented to both region- and object-oriented structural similarity indexes, and it can effectively respond to the structural similarity between two image collections.

### 4.4. Comparison Experiments

To reflect the advantage of RMTF-Net, we trained seven state-of-the-art models, including Unet [14], Segnet [48], AttUnet [35], TransUnet [42], TransFuse [43], FAnet [49], and SSFormer [50] for contrast. The experiments of models used the same datasets. All parameters of the comparison experiments were set to default values. The following experimental results are presented in terms of datasets.

#### 4.4.1. LGG Dataset

Quantitative Evaluation: From Table 1 we can observe that, on the LGG dataset, the proposed model outperforms other methods in all metrics. Compared with AttUnet, SSFormer, TransFuse, FAnet, Unet, Transunet, and Segnet, our model improves 2.8%, 1.0%, 4.4%, 0.3%, 1.3%, 0.9%, 10.4% on MeanDice and 4.6%, 1.6%, 7.1%, 0.3%, 2.1%, 1.3%, 16% on MeanIoU, respectively. After the addition of the GFI module, the local and global information of the features extracted from the encoder part is balanced. This makes RMTF-Net 0.9% and 0.4% higher in Sm and Em scores, respectively, than SSFormer without the addition of this class of modules. Thus, it is evident that the predictions of the proposed model are most similar to the ground truth.

**Figure 4:**
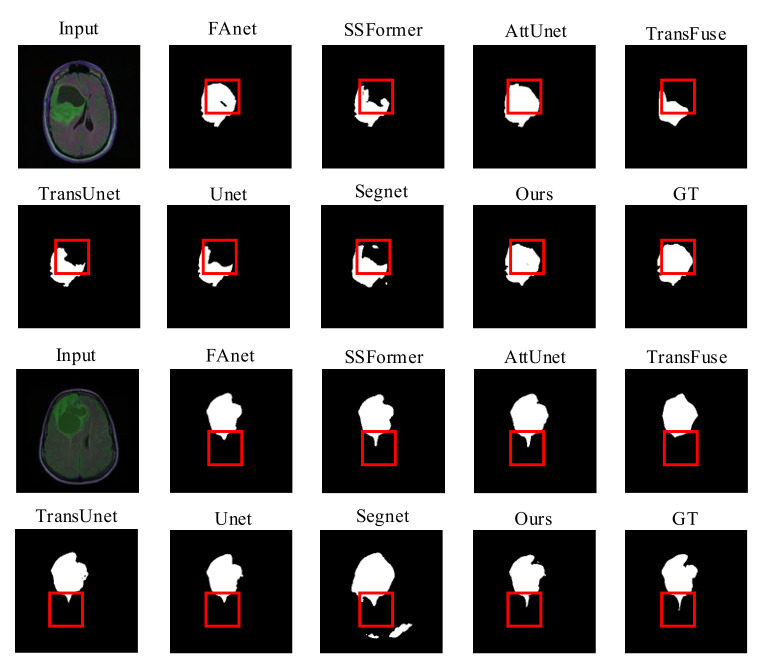
Quality results on the LGG dataset.

Quality Evaluation: Figure 4 displays visual comparisons of the proposed model’s comparison experiment using the LGG dataset. The proposed network utilizes a GFI module in the encoder part, which allows global attention features to be incorporated into the skip connection feature maps. This action can prevent the loss of some fine details during the restoration process due to simple up-sampling and limit the interference of irrelevant background feature information with the segmentation operation.

The AttUnet also applies an attention gate (AG) module to introduce an attention mechanism and enrich the skip connection feature maps used for feature fusion. Thus, other than AttUnet and RMTF-Net, the rest of the mods exhibit varying degrees of target losses in the necrotic parts of the glioma denoted by the red boxes, as seen in the comparison images in rows 1 and 2 of Figure 4. The RMTF-Net focuses on convolutional blocks with residual connections in the encoder, which lifts the performance of the network as well as the feature extraction ability of the encoder. Consequently, the segmentation of the detailed boundary part of the glioma in the left outer part of the red box is significantly better than that of the AttUnet. Moreover, in the region marked by the red box in the comparison images shown in the third and fourth rows of Figure 4, RMTF-Net shows superior performance over other networks in the segmentation of small crab foot variations in gliomas. Segnet does not introduce a global attention mechanism in the encoding process, making it unable to dispense well with the interference of background information with semantic information, so that mis-segmentation occurs in the experiments shown in lines 1 and 2 of Figure 4. In contrast, RMTF-Net does not show this phenomenon.

#### 4.4.2. BraTS2019 Dataset

Quantitative Evaluation: As shown in Table 2, on the Brats2019 dataset, RMTF-Net significantly outperforms all methods except SSFormer in all metrics, where MeanDice and MeanIoU reach 0.821 and 0.743, respectively. Compared with AttUnet, Unet, and Segnet, our model improves in MeanDice by 9.3%, 1.3%, and 4.3%, respectively, which shows the superiority of RMTF-Net in the transformer. SSFormer uses PVTv2 as the backbone, which has a stronger global feature extraction capability compared with the mix transformer applied in RMTF-Net. Therefore, its Em metric is slightly higher than RMTF-Net in the performance of the BraTS2019 dataset with more global features. Nevertheless, the Em metric of RMTF-Net also has a significant improvement compared with other models, and the rest of the metrics are higher than SSFormer, which is enough to prove the advancement of RMTF-Net. In comparison with these current networks, TransUnet and RMTF-Net lead in MeanIoU by nearly two percentage points, which indicates that after introducing overlapped patch embedding, the boundary information of chunks is well-protected.

**Figure 5:**
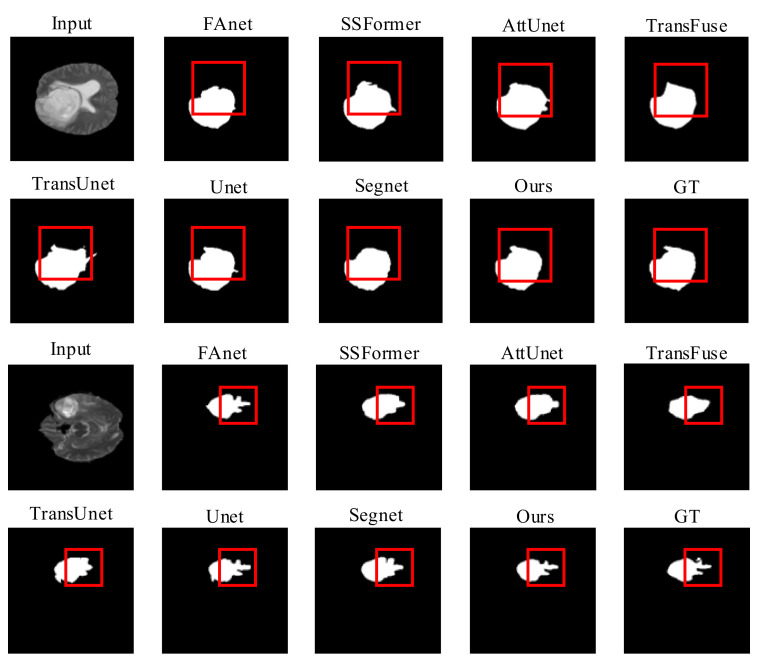
Quality results on the BraTS2019 dataset.

Quality Evaluation: Figure 5 shows visual comparisons of the BraTS2019 dataset of the proposed model with a contrast experiment. Due to the use of overlapped patch embedding in MiT, RMTF-Net has a robust ability to acquire detailed features at the target edges. The two comparison studies in Figure 5 show that RMTF-Net greatly outperforms TransUnet, SSFormer, and TransFuse in edge-complex segmentation tasks, which use the transformer structure with non-overlapped patch embedding. We can also observe that the segmentation results of RMTF-Net in the two sets of experiments are better than all the other state-of-the-art models and are closer to the results of manual segmentation by doctors. Specifically, due to TransFuse’s over-focus on global features, some local features are lost. Therefore, TransFuse has a blurred boundary in the second set of comparison experiments in Figure 5. However, due to the use of a local–global balanced feature during encoding, RMTF-Net has a clear boundary.

#### 4.4.3. BraTS2020 Dataset

Quantitative Evaluation: As shown in Table 3, RMTF-Net improvs the MeanDice, MeanIoU, wFm, and Sm metrics by 0.8%, 0.9%, 1.0%, and 0.5%, respectively, over thesuboptimal model on the BraTS2020 dataset. The MeanIoU and meanEm metrics of the proposed model in this paper showed significant increases compared with AttUnet, TransFuse, Transunet, and Segnet, and MeanDice and MeanIoU improved by up to eight and twelve percentage points, respectively. After analysis, the reason for this advantage may be that in the past network, the relevance of contextual information is reduced because of a large number of convolution and pooling operations; after adding the GFI module, the contextual information in the RMTF-Net is fed promptly and the above information can be sufficiently utilized. The Em metric of TransFuse is slightly higher than ours by 0.7%. Our analysis suggests that this is since, after incorporating local and global features from CNNs and transformer, TransFuse uses the Attention Gate structure to further enhance the global attention features. However, except for the Em metric, all other metrics of RMTF-Net are higher than TransFuse on this dataset, which is enough to show the advancement of our model. Among the models used in the experiments, the model proposed achieves the best results for medical image segmentation.

**Figure 6:**
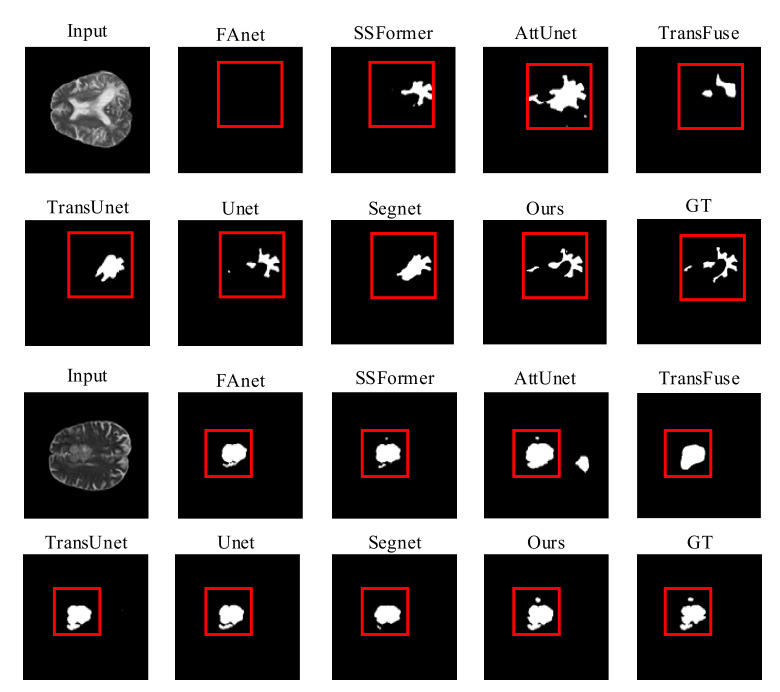
Quality results on the BraTS2020 dataset.

Quality Evaluation: Figure 6 shows visual comparisons of the BraTS2020 dataset of the proposed model with state-of-the-art methods. Except for RMTF-Net, which produces a more accurate segmentation, we can plainly see that the segmentation of the models in the comparison trials presented in the photos in rows 1 and 2 of Figure 6 is not sufficient, and FAnet fails to even detect the segmentation target. Because of the serial use of transformers and CNNs in TransUnet, the transformers will have difficulty obtaining global attention features from the tiny feature maps extracted by CNNs. As a result, it performs poorly at keeping background information from obstructing the segmentation job, which causes a semantic loss in the comparison experiments represented by the images in the third and fourth rows of Figure 6. In both sets of comparison trials presented in Figure 6, the likelihood of identifying the most difficult to segment regions by the naked eye is extremely limited. Nevertheless, the RMTF-Net fused with the use of transformers and CNN is still able to segment the discontinuous point-like fine tumors. As a result, RMTF-Net is able to sample with high accuracy and has excellent interference resistance. In contrast to SSFormer, the mix transformer used by RMTF-Net can better protect the edge information of the target, so in the experiments shown in rows 3 and 4 of Figure 6, SSFormer has blurred edges and RMTF-Net has sharpened edges.

### 4.5. Ablations Experiments and Analysis

#### 4.5.1. Effectiveness of GFI Module and Hybrid Loss

To further analyze the impact of the GFI module and hybrid loss module in the proposed model on the overall performance of the model, we compare the performance of the three models. Table 4 shows the comparative results of MeanDice and MeanIoU scores of these variants on the three datasets. Where backbone represents the remaining network model of RMTF-Net after removing the GFI module and hybrid loss modules, w/H-loss indicates the backbone model with the hybrid loss module added, and w/GFI means the backbone model with the GFI module added.

We can observe that the network with the addition of the GFI module alone brings a significant performance improvement in terms of MeanDice and MeanIoU. To be precise, the network with the GFI module alone improves the MeanDice and MeanIoU scores by about one percentage point on all three datasets. The network with the hybrid loss module alone performed less well, slightly worse than the original backbone network in terms of MeanDice and MeanIoU on the BraTS2019 dataset and MeanIoU score on the BraTS2020 dataset, but slightly better in all other evaluation metrics. This clearly demonstrates the effectiveness of both the GFI module and hybrid loss modules. After further experimentation, we find that the segmentation accuracy could be maximized after using these two modules in a fusion. It is worth mentioning that on the BraTS2019 dataset, the network fusing the two modules improved by 1.3% and 2.2% on MeanDice and MeanIoU, respectively. The optimal scores are achieved on all three datasets.

#### 4.5.2. Size of the MiT

In order to determine the better MiT size, we experimentally analyze both large and base MiT module sizes. During the experiment, we ensure that all other variables are the same. The experimental results are shown in Table 5. The variant named RMTF-Net-L uses the large MiT as a component, while RMTF-Net-B uses the base one. We can observe that the “RMTF-Net-B” model achieves optimal results for MeanDice and MeanIoU scores on all three datasets. Concretely, the “RMTF-Net-B” model leads by 1.1%, 1.0%, and 0.4% in MeanIoU scores for LGG, BraTS2019, and BraTS2020 datasets, respectively. It can lead to a better segmentation effect with low computational power overhead, so we finally choose the “RMTF-Net-B” model for the size of the MiT model.

#### 4.5.3. Effect of Different Transformer Structures

To find the best transformer encoder structure, we chose four recently popular transformers for experimental comparison, namely poolformer [51], PVT, pyramid vision transformer v2 (PVT_v2) [52] and mix transformer. We have designed four variants: PoolTF-Net, PTF-Net, PTv2F-Net, and RMTF-Net, corresponding to the use of poolformer, PVT, PVTv2, and MiT structures, respectively. As shown in Table 6, RMTF-Net achieves the best results on both MeanDice and MeanIoU on the three datasets. Meanwhile, the other three variants achieve the second-best results on the LGG, BraTS2019, and BraTS2020 datasets, respectively. It is obvious that the mix transformer has better robustness and stronger generalization ability and remains in the lead for extracting features.

#### 4.5.4. Effectiveness of Global–Local Feature Fusion for Segmentation Tasks

To deeply explore the effectiveness of global–local feature fusion for segmentation tasks, we compared the performance of three models. Table 7 shows the comparative results of MeanDice and MeanIoU scores of these variants on the three datasets. The MiTencoder-Net and RCNNencoder-Net variants remove the GFI module, and MiTencoder-Net utilizes only MiT as the encoder, while RCNNencoder-Net employs RCNN only. In the RCNN, the features extracted gradually change from only local to only global as the network deepens. On the contrary, the mix transformer changes the extracted features from only global to only local as the network deepens. In general, these two variants do not perform global–local feature fusion operations during the encoding process. As shown in Table 7, we can clearly observe that RMTF-Net achieves the best results on both MeanDice and MeanIOU for the three different datasets. On the LGGS dataset, RMTF-Net outperforms the two variant models on MeanDice and MeanIOU by 0.6%, 0.8% and 0.7%, 1.0%, respectively. Meanwhile, MiTencoder-Net is superior to RCNNencoder-Net on the BraTS2019 and BraTS2020 datasets, and RMTF-Net outperforms MiTencoder-Net by 0.9%, 1.5% and 1.7%, 1.8% in the MeanDice and MeanIOU metrics, respectively. The experimental results display that global–local features fusion is effective and that not fusing global and local features during the encoding of the network leads to poor segmentation results.

## 5. Conclusions

In this paper, we sought to solve challenges in the segmentation of brain tumors such as complex background, discontinuous point-like fine tumors segmentation, and complicated boundary information. We proposed an advanced segmentation method, namely RMTF-Net. It consists of a residual MiT encoder and a feature decoder. In the residual MiT encoder, we adopt an MiT to reduce the impact of complex background information on segmentation tasks. Benefiting from the overlapped patch embedding applied in MiT, the boundary information is protected, which leads to a strong ability in the boundary encoding of the network. Due to the parallel fusion strategy, we fused the MiT and RCNN in the residual MiT encoder so that the encoder can obtain a local–global balanced feature for encoding at each step to obtain quality features. In the feature decoder, we proposed a GFI module to enrich the context with the global attention feature provided by MiT, which can avoid the loss of some fine details via simple up-sampling during the decoding process. Experimental results on three datasets demonstrate that RMFT-net has better performance in brain tumor segmentation compared with some state-of-the-art models. Moreover, the visual comparisons of three datasets show RMTF-Net greatly outperforming in the brain tumor segmentation task. The limitation of this study is that the proposed method only deals with 2D images. Moreover, we only explored the performance of our model on brain tumor segmentation tasks. In the future, we will extend the method to segment 3D images and apply this method to other segmentation tasks.

## Figures and Tables

**Table 1:** The quantitative result on the LGG dataset (bold numbers indicate the best performance).

Dataset	Method	MeanDice	MeanIoU	wFm	Sm	Em
LGG	AttUnet [35]	0.907	0.836	0.892	0.922	0.975
	SSFormer [50]	0.925	0.866	0.926	0.939	0.984
	TransFuse [43]	0.891	0.811	0.892	0.915	0.977
	FAnet [49]	0.932	0.879	0.934	0.945	0.987
	Unet [14]	0.922	0.861	0.925	0.937	0.983
	Transunet [42]	0.926	0.869	0.928	0.940	0.986
	Segnet [48]	0.831	0.722	0.790	0.858	0.928
	RMTF-Net (Ours)	**0.935**	**0.882**	**0.941**	**0.948**	**0.988**

**Table 2:** The quantitative result on the BraTS2019 dataset (bold numbers indicate the best performance).

Dataset	Method	MeanDice	MeanIoU	wFm	Sm	Em
BraTS2019	AttUnet [35]	0.728	0.622	0.703	0.816	0.869
	SSFormer [50]	0.820	0.735	0.821	0.877	**0.942**
	TransFuse [43]	0.804	0.720	0.808	0.873	0.933
	FAnet [49]	0.780	0.699	0.786	0.858	0.899
	Unet [14]	0.808	0.727	0.814	0.874	0.928
	Transunet [42]	0.755	0.659	0.753	0.838	0.912
	Segnet [48]	0.778	0.69	0.781	0.856	0.910
	RMTF-Net (Ours)	**0.821**	**0.743**	**0.831**	**0.883**	0.933

**Table 3:** The quantitative result on the BraTS2020 dataset (bold numbers indicate the best performance).

Dataset	Method	MeanDice	MeanIoU	wFm	Sm	Em
BraTS2020	AttUnet [35]	0.730	0.613	0.692	0.808	0.869
	SSFormer [50]	0.810	0.724	0.815	0.875	0.937
	TransFuse [43]	0.806	0.714	0.808	0.872	**0.948**
	FAnet [49]	0.804	0.718	0.812	0.870	0.932
	Unet [14]	0.807	0.724	0.815	0.874	0.928
	Transunet [42]	0.756	0.659	0.757	0.839	0.911
	Segnet [48]	0.784	0.693	0.791	0.858	0.924
	RMTF-Net (Ours)	**0.818**	**0.733**	**0.825**	**0.880**	0.941

**Table 4:** Result of the effectiveness of the GFI module and hybrid loss (Bold numbers indicate the best performance).

Variants	Module		Dataset					
	GFI Module	Hybrid Loss	LGG		BraTS2019		BraTS2020	
			Mean Dice	Mean IoU	Mean Dice	Mean IoU	Mean Dice	Mean IoU
backbone			0.929	0.873	0.810	0.734	0.813	0.726
w/H-loss		√	0.93	0.874	0.804	0.725	0.808	0.721
w/GFI	√		0.931	0.877	0.818	0.738	0.815	0.729
RMTF-Net	√	√	**0.935**	**0.882**	**0.821**	**0.743**	**0.818**	**0.733**

**Table 5:** Result of the size of the MiT experiment (bold numbers indicate the best performance).

Variants	Dataset					
	LGG		BraTS2019		BraTS2020	
	Mean Dice	Mean IoU	Mean Dice	Mean IoU	Mean Dice	Mean IoU
RMTF-Net-L	0.927	0.871	0.815	0.733	0.814	0.729
RMTF-Net-B	**0.935**	**0.882**	**0.821**	**0.743**	**0.818**	**0.733**

**Table 6:** Result of the effect of different transformer structures experiment (bold numbers indicate the best performance).

Variants	Dataset					
	LGG		BraTS2019		BraTS2020	
	Mean Dice	Mean IoU	Mean Dice	Mean IoU	Mean Dice	Mean IoU
PoolTF-Net	0.932	0.877	0.805	0.725	0.813	0.727
PTF-Net	0.934	0.882	0.806	0.725	0.811	0.726
PTv2F-Net	0.929	0.873	0.813	0.729	0.802	0.714
RMTF-Net	**0.935**	**0.882**	**0.821**	**0.743**	**0.818**	**0.733**

**Table 7:** Result of the effectiveness of global–local feature fusion for segmentation tasks. (bold numbers indicate the best performance).

Variants	Dataset					
	LGGS		BraTS2019		BraTS2020	
	Mean Dice	Mean IoU	Mean Dice	Mean IoU	Mean Dice	Mean IoU
MiTencoder-Net	0.929	0.874	0.812	0.728	0.801	0.715
RCNNencoder-Net	0.928	0.872	0.810	0.734	0.808	0.723
RMTF-Net	**0.935**	**0.882**	**0.821**	**0.743**	**0.818**	**0.733**

## Data Availability

The datasets are provided by BraTS 2019 Challenge, BraTS 2020 Challenge and are allowed for personal academic research. The specific link to the dataset is https://ipp.cbica.upenn.edu/ (accessed on 20 August 2022). And the LGG dataset is obtained from Kaggle and the link to the dataset is https://www.kaggle.com/datasets/mateuszbuda/lgg-mri-segmentation (accessed on 20 August 2022).
